# Concurrent and Simultaneous Polydrug Use: Latent Class Analysis of an Australian Nationally Representative Sample of Young Adults

**DOI:** 10.3389/fpubh.2013.00061

**Published:** 2013-11-28

**Authors:** Lake-Hui Quek, Gary C. K. Chan, Angela White, Jason P. Connor, Peter J. Baker, John B. Saunders, Adrian B. Kelly

**Affiliations:** ^1^Faculty of Health Sciences, Centre for Youth Substance Abuse Research, The University of Queensland, Brisbane, QLD, Australia; ^2^School of Medicine, The University of Queensland, Brisbane, QLD, Australia; ^3^Faculty of Health Sciences, School of Population Health, The University of Queensland, Brisbane, QLD, Australia

**Keywords:** young adults, polydrug use, latent class analysis, cluster, risk and protective factors, simultaneous

## Abstract

**Background**: Alcohol use and illicit drug use peak during young adulthood (around 18–29 years of age), but comparatively little is known about polydrug use in nationally representative samples of young adults. Drawing on a nationally representative cross-sectional survey (Australian National Drug Strategy Household Survey), this study examines polydrug use patterns and associated psychosocial risk factors among young adults (*n* = 3,333; age 19–29).

**Method**: The use of a broad range of licit and illicit drugs were examined, including alcohol, tobacco, cannabis, cocaine, hallucinogens, ecstasy, ketamine, GHB, inhalants, steroids, barbiturates, meth/amphetamines, heroin, methadone/buprenorphine, other opiates, painkillers, and tranquilizers/sleeping pills. Latent class analysis was employed to identify patterns of polydrug use.

**Results**: Polydrug use in this sample was best described using a 5-class solution. The majority of young adults predominantly used alcohol only (52.3%), alcohol and tobacco (34.18%). The other classes were cannabis, ecstasy, and licit drug use (9.4%), cannabis, amphetamine derivative, and licit drug use (2.8%), and sedative and alcohol use (1.3%). Young adult males with low education and/or high income were most at risk of polydrug use.

**Conclusion**: Almost half of young adults reported polydrug use, highlighting the importance of post-high school screening for key risk factors and polydrug use profiles, and the delivery of early intervention strategies targeting illicit drugs.

## Introduction

Young adulthood (18–29 years) is a high risk time for the use and misuse of licit and illicit substances, relative to other age groups (1). Alcohol is the most common drug used (86%) in this group, with 55% of young adult Australians drinking at levels that are at risk of alcohol-related injury arising from a single occasion in the past month (2). Though decreasing over recent decades, approximately 24% of young Australians smoke tobacco (2). The use of illicit drugs peaks in young adulthood, with rates two to three times higher than that of the general population (2). The use and misuse of individual drugs are associated with substantial costs to individuals, communities, and health systems (3–6).

In recent years there has been increased research attention on patterns of polydrug use [defined as the consumption of more than one drug during a specific time period; (7)] among young adults. Much of this research has been done on relatively small convenience samples (e.g., street-based, drug injecting, emergency room, and club patrons), where multiple drugs are frequently used consecutively or simultaneously, often for their perceived counteracting or complementary effects (8–15). These targeted studies are important because they highlight the near universal patterns of polydrug use that occur in specific populations (9, 10), and the substantially higher risks to mental and physical health faced by polydrug users compared to single drug users (16–18). However, polydrug use in specific populations tells us little about rates of polydrug use in wider populations.

Comparatively few studies have examined young adult polydrug use based on large scale population-based samples. Extant research has been conducted on younger Australians [less than 18 years; (19)], Latin Americans [mean age 35; (20)], adult American populations [mean age 46; (16)], adult populations from Great Britain [mean age 43 years; (21)], and large scale Australian twin registry studies [mean 30 years; (17)]. Among these studies, there is variation in the window of time used to define polydrug use. Some studies have defined polydrug use as the use of more than one drug in a lifetime (16, 17, 19), in the past year (21), and in the past month (20). The narrower the window of time used to define polydrug use, the closer the potential proximity of use of different drug types. However, in large scale population studies, this results in smaller cell sizes for individuals reporting specific combinations of drug use, and this creates challenges for established procedures like latent cluster analysis, which rely on minimum cell sizes to reliably detect clusters. In part because of these statistical power challenges, and the inherent challenges in assessing temporally linked drug taking events in large scale surveys, most such studies utilize wide windows of time (most commonly lifetime prevalence) to detect polydrug use.

The first goal of the present study was to evaluate the nature and extent of polydrug use in a nationally representative population of Australian young adults (18–29 years of age) using two temporal windows to define polydrug use. The first was polydrug use in the previous year [hereafter termed *concurrent polydrug use*, consistent with earlier research; (22)], and the second was *simultaneous use*, a dimension rarely assessed in large scale population studies. One-year temporal windows for assessing polydrug use have the advantage of capturing developmentally sensitive periods, relative to lifetime prevalence, but are likely to retain statistical power to determine clusters, relative to shorter temporal windows. Simultaneous use potentially yields an understanding of how various combinations of drugs are used for their synergistic properties including the enhancement of specific effects and the suppression of unwanted effects ones (23, 24). A key concern about polydrug use is that the effect of individual drugs are often compounded, and harmful physiological effects accumulate in the body (23–26). We sought to describe the extent to which simultaneous use characterized usage patterns in young adults with recent experience in the use of different substances. The second goal of this study was to examine demographic and broad socio-emotional correlates of polydrug use clusters. Compared to adolescence, the lives of young adults often involve increased disposable income, heightened social independence, and fewer societal restrictions on the use of certain drugs (27, 28). In this study we explored the association of drug use clusters with socioeconomic status (29, 30), educational level, peer substance use (31), and mental health (16, 21).

The present study was based on the National Drug Strategy Household Survey (NDSHS), which is unique in that it is one of the few nationally representative studies that enables an examination of polydrug clusters, and an exploration of simultaneous drug use. The study employs latent class analysis (LCA), which is a probabilistic method of determining the size and structure of drug use profiles. This analytic method has rarely been used in large scale population research on young adults, but has been extensively used with other age groups (16, 17, 21). The study is also a rare examination of how a comprehensive range of drugs, including alcohol and tobacco, cluster together in young Australians.

## Materials and Methods

### Sample

The sample consisted of 3,333 participants in the 2007 NDSHS (32) who were aged between 19 and 29 years (42.2% males, mean age = 23.78, SD = 3.42). Of this initial sample, 322 (9.67%) participants were excluded due to non-response for any of the drug-related items and four participants were excluded as they reported using a fictitious drug. The final sample size for the LCA was 3011. Among this sample, 292 participants had one or more missing data in the covariates. Full information maximum likelihood estimation was used to handle the missing data. A supplementary analysis with complete cases only was conducted to evaluate the robustness of the results.

#### Measures

##### Concurrent drug use

This measure was based on NDSHS drug use frequencies for a broad range of drugs used in the previous 12 months. Tobacco use was assessed with the item “How often do you now smoke cigarettes, pipes, or other tobacco products?” with responses of “daily,” “at least weekly,” “less often than weekly,” “not at all but I have smoked in the last 12 months,” and “not at all and I have not smoked in the last 12 months.” These responses were recoded as 0 “No smoking in the last 12 months” and 1 “Smoking in last 12 months.” Alcohol consumption was measured using a single, yes/no response item: “Have you had an alcoholic drink of any kind in the last 12 months?” Yes/no response items were also used to assess for use of painkillers, tranquilizers/sleeping pills, steroids, barbiturates, and meth/amphetamine: “Have you used (drug type) for non-medical purpose in the last 12 months?” Consumption of all other drugs, including cannabis/marijuana, heroin, methadone/buprenorphine, cocaine, hallucinogens, ecstasy, ketamine, GHB, and inhalants, was assessed using yes/no questions: “Have you used (drug type) in the last 12 months?”

##### Simultaneous drug use

Simultaneous drug use was examined in a separate series of questions in which respondents reported using a drug in the past 12 months (except tobacco and alcohol), using the question “Which of the following did you use at the same time, on at least one occasion that you used (drug type)?” With a list of possible drugs (includes alcohol but not tobacco). This question identified pairs of drugs that are being used simultaneously at least once in the past 12 months [similar to previous studies, e.g., Ref. (26, 33, 34)]. To increase reliability of simultaneous drug use coding, it was coded as present only when (i) both drugs were used in the past 12 months, and (ii) simultaneous use were also reported in both drug types (e.g., amphetamines being used simultaneously with cannabis, and cannabis being used simultaneously with amphetamines).

##### Demographic and psychosocial correlates

The 10-item Kessler Psychological Distress Scale (35) was used to measure depressive and anxiety symptoms in the past 4 weeks [e.g., about how often did you feel tired out for no good reason? (5-point scale ranging from 1 “None” to 5 “All,” alpha = 0.86)]. Peer drug use was accessed with the item “About what proportion of your friends and acquaintances use any of the following? Alcohol/Tobacco/Cannabis” (same 5-point response scale), and was recoded into 0 “Less than half” or 1 “Half or more.” General health was measured with the item “In general, would you say your health is …” (1 “Excellent” to 5 “Poor”). Due to the low frequency of participants reporting poor health (*n* = 35, 1.16% of the analysis sample), the categories “fair” and “poor” were combined to form a single category. Demographic variables included in the analysis were: sex (0 “male,” 1 “female”); marital status (1 “never married,” 2 “widowed/divorced/separated,” 3 “married/defacto”); employment status (1 “employed/self-employed,” 2 “unemployed,” 3 “home duties,” 4 “student,” 5 “retired/unable to work/other”); high school completion (0 “completed,” 1 “not completed”); income levels (0 “$41,600 or above,” 1 “$13,000–41,599,” 2 “$12,999 or below,” 3 “prefer not to say/don’t know”); socioeconomic indices for areas (SEIFA, 1 “Least advantaged/1st quartile” to 2 “Most advantaged/fourth quartile” = 4), and regionality (1 “major cities,” 2 “inner regional,” 3 “outer regional,” 4 “remote/very remote”) (36).

#### Procedure

There were 23,356 respondents from households across all states and territories of Australia who participated in the NDSHS and 3,333 were in the targeted age range (between 19 and 29 years). Participants were randomly selected using a stratified design based on statistical local areas (36). The survey involved two modes of delivery – drop and collect (19,818 respondents) and computer-assisted telephone interview (CATI) (3,538 respondents). For each household, the respondent was the household member aged >12 years whose birthday was next to occur in the family. Further information on survey procedures is available in AIHW reports (37). The survey was approved by the Australian Institute of Health and Welfare Ethics Committee. Informed consent was obtained from all subjects. Access to these survey data was approved by the Australian Social Science Data Archive and by The University of Queensland Human Research Ethics Committee.

#### Analysis

To identify patterns of polydrug use, LCA was performed on the last year use of nine drugs: alcohol, tobacco, cannabis, ecstasy, meth/amphetamine, pain-killer, tranquilizers/sleeping pills, cocaine, and hallucinogens. LCA is a technique that identifies sub-classes within a large population based on similarity of response to a set of measured variables. This technique is characterized by two sets of parameters: (1) The estimated proportion of each class in the population, and (2) the probability of an individual in a particular class using a certain drug.

Determination of number of classes was based on a number of fit criteria (38). First, the Bayesian Information Criterion (BIC) (39), Sample Size Adjusted Bayesian Information Criterion (SSABIC) (40), and the Akaike Information Criterion (AIC) (41) were compared across models with different number of classes. A lower value of these information criteria indicates a better balance model parsimony and model fit. Second, the Lo–Mendell–Rubin adjusted likelihood ratio test (42) was used to compare the fit of a model with *k* class with a model with *k-1* class. A significant *p*-value (*p* < 0.05) from this test indicates a *k* class model fits the data better than a *k-1* class model. Third, the number of significant bivariate residuals was used to access the validity of the local independent assumption of LCA. A large number of significant bivariate residuals indicates that this assumption is severely violated. Finally, the average posterior probabilities were used to evaluate the classification quality. Model fitting began with a one-class solution, and the number of classes was increased successively up to a 6-class solution. Once the optimal number of classes was determined, covariates were added to the LCA to examine their associations with the latent class membership. In this study, data was prepared with STATA 11 (43) and analyses were performed with Mplus 6.01 (44).

## Results

### Latent class analysis

Model fit statistics for 1–6-class solutions are presented in Table 1. A 3-class solution attained the lowest value of BIC, and a 5-class solution attained the lowest value of AIC and SSABIC. Results from the LMR-LRT suggested that the fit of the 4-class model was significantly better than a 3-class model but not worse than the 5-class model. Simulation suggested that the performance of SSABIC and LMR-LRT were similar and superior to other statistics criteria (45, 46). SSABIC suggested the 5-class solution was optimal but LMR-LRT suggested the 4-class solution was optimal. Since they pointed to different solutions and other fit statistics of these two solutions were very close, both were examined on the basis of interpretability (44). The 4-class solution identified a class with cannabis, amphetamine derivatives, and licit drug use but the 5-class model subdivided this class into two – a class with cannabis, amphetamine derivatives, and licit drug use and a class that primarily used sedatives and alcohol. This classification was consistent with previous findings (26). The 5-class solution was chosen as the optimal solution as it yielded classification that was clearly distinct and interpretable, and had adequate class sizes with high average posterior probabilities (>0.80).

**Table 1:** **Fit statistics from 1 to 6-class models**.

Class	Likelihood	df	AIC	BIC	SSABIC	*p*-Value (LMR-LRT)	No. of Sig. residuals
1	−7969.24	9	15956.47	16010.55	15981.95	N/A	98
2	−6859.55	19	13757.09	13871.26	13810.89	0.00	31
3	−6718.08	29	13494.15	13668.40	13576.26	0.00	4
4	−6683.10	39	13444.20	13678.54	13554.60	0.01	0
**5**	−**6658.02**	**49**	**13414.03**	**13708.46**	**13552.77**	**0.14**	**0**
6	−6644.71	59	14307.41	13761.93	13574.46	0.15	1

Bolded text indicates the chosen model based on outlined criteria.

**Figure 1:**
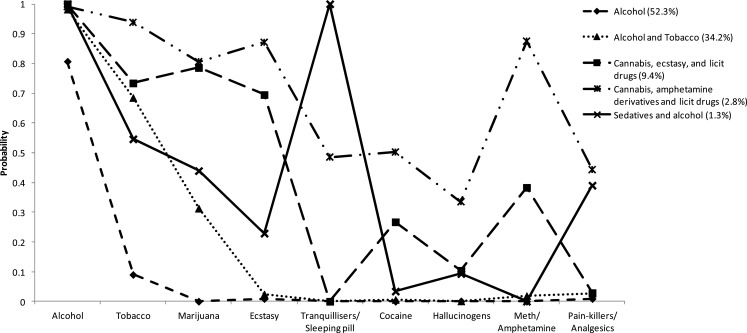
**Probabilities of using each drug type (in last 12 months) for the five classes identified in the latent class analysis**.

Each of the five classes was described below using the probabilities of drugs use in the past 12 months (see Figure 1). For heuristic purposes, the nomenclature adopted for each class is based on the type and range of substances with posterior probabilities greater than 0.65.

Class 1: Participants in this class were predominantly alcohol users (0.80 probability of alcohol use), with a small probability (0.10) of tobacco use and nearly zero probabilities of other drug use. This class was labeled *Alcohol only*, and the prevalence estimate of this class was 52.3% (*n* = 1572).
Class 2: These participants reported universal alcohol use (0.98), high probability of tobacco use (0.66), moderate probability of cannabis use (0.31), and negligible probabilities of other drug use (below 0.05). This class was labeled *Alcohol* and *tobacco*, and the prevalence estimate of this class was 34.18% (*n* = 1028).
Class 3: Participants in this class universally reported using alcohol (1.00), very high probabilities of tobacco, cannabis, and ecstasy use (probabilities 0.70 or higher), moderate probability of meth/amphetamine and cocaine use (0.22–0.38) and low probabilities of hallucinogens and other drugs. This class was labeled *Cannabis*, *ecstasy*, and *licit drug use*, and the prevalence estimate of this class was 9.44% (*n* = 284).
Class 4: This class was characterized by universal use of alcohol (0.99), very high probabilities of tobacco, cannabis, ecstasy, and meth/amphetamine use (0.70–0.89) and moderate to high probabilities of all other drug use. This class was labeled *Cannabis*, *amphetamine derivatives*, and *licit drug use*, and the prevalence estimate of this class was 2.79% (*n* = 83).
Class 5: These participants reported universal alcohol and tranquilizer use (1.00), moderate to high probabilities of tobacco, cannabis, ecstasy, and pain-killer/analgesic use (0.23–0.54), and low probabilities of using other drugs (below 0.10). This class was labeled *Sedative* and *alcohol use*, and the prevalence estimate of this class was 1.31% (*n* = 39).

Table 2 shows the odd ratios and the associated 95% confidence intervals of covariates for class membership. Relative to the *Alcohol* class, being male, being older, not completing high school and having more income were significantly associated with membership in the *class 2* (*alcohol* and *tobacco*), *class 3* (*cannabis*, *ecstasy*, and *licit drug use*), and *class 4* (*cannabis*, *amphetamine derivatives*, and *licit drug use*) (*p*s < 0.05). Although being older was significantly associated with *class 5* (*Sedatives* and *alcohol*), sex, high school completion, and having more income were not. Young adults without partners and not living in the regional areas were also significant predictors of *class 3* and *class 4* membership. Individuals living in high socioeconomic status areas or those where were unemployed were significantly more likely to be in *class 3* (*p* < 0.05).

**Table 2:** **Relative risk ratio estimates and 95% confidence intervals from the multinomial logistic regression with the Alcohol class as the reference group**.

	Class 2: alcohol and tobacco		Class 3: cannabis, ecstasy, and licit drug use		Class 4: cannabis, amphetamine derivatives, and licit drug use		Class 5: sedatives and alcohol	
	RRR^a^	95% CI	RRR^a^	95% CI	RRR^a^	95% CI	RRR^a^	95% CI
Age
Continuous years	1.03*	(1.01–1.05)	1.07**	(1.03–1.12)	1.10*	(1.02–1.18)	1.14*	(1.01–1.28)
Sex (Ref: Male)
Female	0.83**	(0.73–0.94)	0.69**	(0.54–0.89)	0.39***	(0.25–0.61)	0.96	(0.50–1.85)
High school (Ref: Completed)
Not completed	1.44***	(1.24–1.68)	1.51**	(1.13–2.01)	2.03**	(1.32–3.11)	1.21	(0.51–2.87)
Marital status (Ref: Non-partnered)
Partnered	0.95	(0.82–1.09)	0.56***	(0.43–0.75)	0.45**	(0.27–0.77)	0.46	(0.21–1.02)
Employment status (Ref: Self-employed/employed for wages)
Students	0.94	(0.77–1.14)	1.09	(0.72–1.63)	1.78	(0.82–3.83)	1.15	(0.33–4.01)
Unemployed	1.25	(0.92–1.69)	2.03*	(1.16–3.57)	1.72	(0.75–3.94)	1.12	(0.23–5.46)
Other	1.02	(0.83–1.26)	0.90	(0.57–1.43)	1.24	(0.59–2.61)	0.36	(0.08–1.69)
Income level (Ref: $41,600 or above)
$13,000–$41,599	0.98	(0.84–1.15)	0.79	(0.59–1.05)	0.60*	(0.36–1.00)	1.14	(0.49–2.67)
$12,999 or below	0.75*	(0.60–0.94)	0.49**	(0.31–0.76)	0.25**	(0.11–0.55)	0.29	(0.05–1.75)
Prefer not to say/don’t know	0.64***	(0.50–0.80)	0.32***	(0.19–0.53)	0.21**	(0.09–0.51)	0.27	(0.05–1.55)
Socioeconomic status of area (Ref: Least advantaged)
2nd quartile	0.94	(0.78–1.12)	1.06	(0.72–1.55)	1.04	(0.53–2.01)	1.01	(0.37–2.74)
3rd quartile	0.96	(0.81–1.14)	1.22	(0.85–1.75)	1.19	(0.65–2.20)	1.33	(0.52–3.38)
4th quartile	1.02	(0.86–1.22)	1.44*	(1.02–2.05)	1.55	(0.86–2.77)	1.33	(0.49–3.64)
Remoteness (Ref: Major cities)
Inner regional	1.07	(0.91–1.26)	0.79	(0.56–1.11)	0.49*	(0.27–0.90)	0.75	(0.30–1.89)
Outer regional	1.04	(0.86–1.27)	0.54***	(0.36–0.81)	0.47*	(0.22–0.99)	1.20	(0.43–3.36)
Remote/very remote	0.85	(0.62–1.15)	0.46*	(0.25–0.86)	0.64	(0.24–1.70)	0.50	(0.07–3.78)
Depressive symptoms
Continuous K10 scores	1.01	(1.00–1.02)	1.01	(0.99–1.04)	1.09***	(1.05–1.12)	1.10***	(1.05–1.16)
General health (Ref: Excellent)
Very good	1.15	(0.98–1.34)	0.94	(0.67–1.34)	1.05	(0.54–2.07)	1.26	(0.45–3.57)
Good	1.54***	(1.30–1.81)	1.55*	(1.10–2.18)	2.29*	(1.21–4.36)	2.53	(0.90–7.12)
Fair/poor	1.60***	(1.23–2.09)	1.61	(0.97–2.66)	1.94	(0.89–4.22)	1.86	(0.46–7.47)
Peer alcohol use (Ref: Less than half)
More than half	1.88***	(1.52–2.34)	3.81***	(2.02–7.18)	15.27***	(4.37–53.33)	1.93	(0.42–8.92)
Peer tobacco use (Ref: Less than half)
More than half	1.88***	(1.66–2.13)	2.02***	(1.55–2.64)	3.65***	(2.14–6.22)	1.29	(0.67–2.50)
Peer cannabis use (Ref: Less than half)
More than half	2.43***	(2.02–2.91)	8.61***	(6.56–11.31)	13.85***	(8.75–21.90)	3.45***	(1.76–6.77)

***p < 0.001; **p < 0.01; *p < 0.05.

^a^RRR, relative risk ratio.

Suboptimal general health was associated with *classes*, *2*, *3*, and *4* (*p*s < 0.05), whilst psychological distress was significantly associated with the *classes 4* and *5* (*p*s < 0.05). Both peer alcohol and tobacco use were predictors of *classes 2*, *3*, and *4* (*p*s < 0.05), while peer cannabis use was associated with classes 2–5 (*p*s < 0.05). Peer alcohol use was a stronger predictor of membership in *class 4* (95% CI: 4.37–53.33) than *class 2* (95% CI: 1.52–2.34) and peer cannabis use was a stronger predictor of membership of *class 4* (95% CI: 8.75–21.90) compared to all other classes.

Since there were missing data in the covariate variables and full information maximum likelihood estimation was used to handle the missing data, a supplementary analysis using participants with complete cases was done. The results were essentially the same – there were only negligible differences in the odds ratios estimates (less than 1% difference in most of the odds ratios). Therefore, the results obtained from full information maximum likelihood estimation were robust.

### Simultaneous drug use

Results for the analysis of simultaneous use (see Table 3) indicated that a large proportion of concurrent users engage in simultaneous drug use, especially combined use of alcohol and cannabis (18.45% of analysis sample). The most common other drug pairs included alcohol – ecstasy (9.54%), alcohol – meth/amphetamine (5.91%), cannabis – ecstasy (4.99%), and alcohol – cocaine (3.69%). The results also showed that a very high proportion of concurrent users of two specific drugs also reported using those specific drugs simultaneously. This was especially notable for alcohol and cannabis, alcohol and ecstasy, and alcohol and meth/amphetamine (greater than 90% of concurrent users were simultaneous users for each of these drug pairs).

**Table 3:** **Percentage of participants reporting concurrent use, simultaneous use, and proportion of concurrent users who were also simultaneous users**.

Top 10 simultaneous drug combinations	% Concurrent use (use of these two drugs at an time in the last 12 months)		% Simultaneous use (use of two specific drugs simultaneously)		Percentage of concurrent users also report simultaneous use (%)
	All (%)	95% CI	All (%)	95% CI	
Alcohol and cannabis	20.72	(19.27, 22.17)	18.45	(17.06, 19.84)	89
Alcohol and ecstasy	10.54	(9.44, 11.64)	9.54	(8.49, 10.59)	91
Alcohol and meth/amphetamine	6.52	(5.73, 7.51)	5.91	(5.07, 6.76)	91
Cannabis and ecstasy	7.42	(6.48, 8.35)	4.99	(4.21, 5.77)	67
Cannabis and meth/amphetamine	5.02	(4.24, 5.80)	4.09	(3.38, 4.80)	81
Alcohol and cocaine	4.16	(3.44, 4.87)	3.69	(3.02, 4.37)	89
Meth/amphetamines and ecstasy	4.69	(3.93, 5.45)	3.23	(2.59, 3.86)	69
Cannabis and cocaine	3.19	(2.56, 3.82)	1.76	(1.29, 2.23)	55
Cocaine and ecstasy	3.09	(2.47, 3.71)	1.63	(1.18, 2.08)	53
Alcohol and painkillers	3.22	(2.59, 3.86)	1.50	(1.06, 1.93)	47

A participant can display more than one combination of polydrug use.

## Discussion

The primary aim of this study was to examine patterns of polydrug use (last 12 months) across a broad range of licit and illicit substances in a nationally representative sample of young adults. Over 25% of young adults in this study reported using drugs other than alcohol in the past 12 months. A 5-class solution resulted, with all classes containing a very high probability of alcohol use, and classes named by the unique feature of each cluster. The classes were: *Alcohol only* (52.3%) *Alcohol* and *tobacco* (34.2%), *Cannabis, ecstasy* and *licit drug use* (9.4%), *Cannabis, amphetamine derivatives*, and *licit drug use* (2.8%), and *Sedatives* and *alcohol use* (1.3%). Additional analysis indicated that when young adults reported concurrent use of drug pairs (in the last 12 months), there was a very high probability (>90%) that the respective drugs were used simultaneously.

While alcohol use was evident in every cluster, the groups showed unique features in both the prominence of particular drug types and the breadth of drugs used. Class 2 was characterized by high probabilities of alcohol and tobacco use. Class 3 was characterized by a limited breadth of drugs used, and the use of two illicit drug types – cannabis and ecstasy. Class 4 was characterized by a similar breadth of drug type use, and was unique in terms of its high probability of meth/amphetamine use. Class 5 was unique in that the breadth of drug types was narrow, and this was the only class to have a high probability of sedative use.

In some earlier research (based on different age groups and from other countries), cluster analytic approaches have tended to identify clusters that primarily vary in the breadth of substance uses (i.e., clusters frequently characterized by minimal, limited, or extended use of different substances). The present findings suggest that, at least in the present sample, variability in high probability substances is limited (between two and five substances, including alcohol), and that several classes are characterized by “spikes” (high and unique probabilities for particular drugs) (see Figure 1). These two observations suggest that polydrug use clusters are not simply groupings based on increasing “deviance” in relation to drug use (i.e., subgroups representing those who have progressively expanded their field of preferred substances). Though beyond the scope of this research, the findings point to the possibility that certain subgroups prefer certain combinations of drugs or that there are restraints or sanctions on the use of particular drugs outside the clusters established in this study. For example, subgroups may have positive and negative attitudes about certain drugs based on their legal status (accounting for the split of alcohol/alcohol and tobacco from other classes), the acceptability of common routes of administration [inhaling versus oral ingestion; (47)], or the perceived instrumental properties of particular drug types (e.g., to increase sociability, reduce anxiety). Further research is needed on mechanisms that may account for clustered use of particular drugs in nationally representative samples. The reasons why particular drugs cluster together may vary across subgroups or within subgroups.

Predictors of class membership (relative to the *alcohol* cluster) indicated consistent and relatively strong odd ratios for sex (being female less likely to be associated with polydrug use and this was significant for all clusters), non-completion of high school (for all except the *Sedative* and *alcohol* class), low income (associated with lower likelihood of polydrug use), and peer use of alcohol/tobacco/cannabis (consistently predicted membership of all clusters). There were weak or inconsistent effects for age, remoteness (though outer regional participants were less likely to be in clusters 3 and 4), marital status, and depressed mood. The odds of cluster membership relative to the alcohol group were generally non-significant for employment status and socioeconomic disadvantage (measured using a community-level government index). The effects for general health were in the expected directions (clusters 2–5 reporting lower health), although poor health ratings mostly did not reach statistical significance. In sum, polydrug use was generally associated with being male, having a very low education (high school not completed), having a high income relative to those on a low income, and having a peer group where the majority use alcohol, tobacco, or marijuana.

The secondary analyses exploring simultaneous use of drugs increased our confidence in the polydrug classes identified in the LCA. The percentage of concurrent users who also use simultaneously was high, depending on drug pair endorsements. In line with previous studies [e.g., Ref. (26, 33, 34)], alcohol was simultaneously used with a wide variety of other drugs (notably cannabis, ecstasy, meth/amphetamine, cocaine; all ≥89%). Less commonly used together were cannabis and cocaine, cocaine and ecstasy, and alcohol and painkillers, though prevalence within concurrent users was still high (47–55%). The high rates of simultaneous use of particular drug pairs points to the possibility that certain drugs are commonly used for their synergistic effects [e.g., stimulants used to reduce the depressant effects of alcohol; (23, 24, 48, 49)], or that craving for one drug type may increase cravings for another (50), or that supply chains increase the probability of expansion into other drug types.

The findings have important implications for prevention and early intervention programs and policies relating to young adult drug use. First, the clustering of alcohol and tobacco for a large minority of the sample points to the possibility that alcohol use is a high risk context for tobacco use (51). Though beyond the scope of this study, there is good evidence that alcohol consumption may erode smoking cessation attempts in young people (52), so smoking prevention programs may benefit from an increased focus on the risks of alcohol use. Second, the findings assist in the relative weighting of prevention strategies based on the prevalence of polydrug classes. It is clear that a substantial minority (approximately 12%) of young adults are concurrent users of several drug types, including cannabis, ecstasy, alcohol, and to a smaller extent meth/amphetamines. At least in Australia, the use of amphetamine derivatives (including ecstasy) is comparatively rare in the teenage years, so a focus on the risks associated with amphetamine derivatives (and simultaneous use of other drugs) upon reaching young adult may be useful in stemming the apparent expansion in the use of these drug types. Third, the proportion of young adults who use sedatives and alcohol is small but meaningful, and reinforces the importance of screening and surveillance strategies to detect vulnerable young adults, and strategies to reduce the inherent risks in combining sedatives and alcohol (Table 3). Finally, young adult males with low education were at elevated risk of poly drug use, pointing to the value of prevention programs instituted prior to the upper levels of high school and programs for young males made available in higher paid working environments.

The use of national surveys such as the NDSHS provides a large sample of cross-sectional overview of drug use in the general population. However, the sampling of individuals with a fixed home address or landline can fail to capture high risk young people, such as those in transient accommodation. This may lead to an underestimation of the actual rates of polydrug use in this group (53, 54). Because this was a cross-sectional survey, no conclusions can be made about mechanisms linking the use of different drugs, or the factors that may underlie cluster membership. It remains unclear whether people in particular polydrug classes have communalities in the development of their drug use profiles, or whether some classes merely represent a continuum of severity. This study did not involve measures of quantity or harm, and it is likely that only a very small proportion of this sample had any form of drug dependence. Therefore, results may not generalize to groups with more significant drug problems. The study relies on self-report, although the confidential and anonymous nature of participation is likely to have reduced response bias. Although the overall sample size of this study was large, the sample sizes in the class “Cannabis, amphetamine, derivatives and illicit drugs” and “sedatives and alcohol” were relatively small and this limited the power to identify covariates that were associated with these two classes. Given the large number of possible permutation of simultaneous drug use, we only examined the prevalence of specific drug pairs. Lastly, due to the limitations of the survey design, we were not able to examine simultaneous use tobacco with other drugs.

## Conclusion

While the sole use of alcohol characterized most people in the sample, a substantial minority of young adults were concurrent users of several drug types. Young adults clustered on unique combinations of alcohol, tobacco, cannabis, ecstasy, meth/amphetamine, and sedatives. Simultaneous use of two substances was characteristic of concurrent users. Peer use of alcohol, tobacco, and cannabis were significant correlates of young adult polydrug use. Young males with low education and/or higher income are a high risk group that warrant a prevention and early intervention focus.

## Author Contributions

Lake-Hui Quek drafted this manuscript under the supervision of Adrian B. Kelly and Angela White. Gary C. K. Chan conducted statistical analyses. Angela White participated in the design of the study and provided feedback at all stages. Jason P. Connor provided conceptual direction and feedback on manuscript drafts. Peter J. Baker supervised the statistical analyses. John B. Saunders participated in the design of the project and provided feedback on manuscript drafts. Adrian B. Kelly supervised the overall project, mentored Lake-Hui Quek in manuscript preparation, and assisted with writing the manuscript.

## Conflict of Interest Statement

The authors declare that the research was conducted in the absence of any commercial or financial relationships that could be construed as a potential conflict of interest.

## References

[1] UNODC World Drug Report 2010. Vienna: United Nations Publication (2010). (Sales No. E.10.XI.13).

[2] AIHW 2010 National Drug Strategy Household Survey Report. Canberra: Australian Institute of Health and Welfare (2011).

[3] Degenhardt L Hall W Extent of illicit drug use and dependence and their contribution to the global burden of disease. Lancet (2012) 379(9810):55–70 10.1016/S0140-6736(11)61138-0 22225671

[4] Fergusson DM Boden JM Horwood LJ The developmental antecedents of illicit drug use: evidence from a 25-year longitudinal study. Drug Alcohol Depend (2008) 96:165–77 10.1016/j.drugalcdep.2008.03.003 18423900

[5] Korhonen T Huizink AC Dick DM Pulkkinen L Rose RJ Kaprio J Role of individual, peer and family factors in the use of cannabis and other illicit drugs: a longitudinal analysis among Finnish adolescent twins. Drug Alcohol Depend (2008) 97(1–2):33–43 10.1016/j.drugalcdep.2008.03.015 18455885 PMC2574687

[6] van Leeuwen AP Verhulst FC Reijneveld SA Vollebergh WAM Ormel J Huizink AC Can the gateway hypothesis, the common liability model and/or, the route of administration model predict initiation of cannabis use during adolescence? A survival analysis-the TRAILS study. J Adolesc Health (2011) 48(1):73–8 10.1016/j.jadohealth.2010.05.008 21185527

[7] Boeri MW Sterk CE Bahora M Elifson KW Poly-drug use among ecstasy users: separate, synergistic, and indiscriminate patters. J Drug Issues (2008) 38(2):517–42 10.1177/002204260803800207 23913981 PMC3729427

[8] Blow FC Walton MA Barry KL Murray RL Cunningham RM Massey LS Alcohol and drug use among patients presenting to an inner-city emergency department: a latent class analysis. Addict Behav (2011) 36(8):793–800 10.1016/j.addbeh.2010.12.028 21514734 PMC4020511

[9] Carlson RG Wang J Falck RS Siegal HA Drug use practices among MDMA/ecstasy users in Ohio: a latent class analysis. Drug Alcohol Depend (2005) 79(2):167–79 10.1016/j.drugalcdep.2005.01.011 16002026

[10] Grov C Kelly BC Parsons JT Polydrug use among club-going young adults recruited through time-space sampling. Subst Use Misuse (2009) 44:848–64 10.1080/10826080802484702 19444726 PMC2683356

[11] Kuramoto SJ Bohnert AS Latkin CA Understanding subtypes of inner-city drug users with a latent class approach. Drug Alcohol Depend (2011) 118(2–3):237–43 10.1016/j.drugalcdep.2011.03.030 21530105 PMC3153580

[12] Lankenau SE Clatts MC Patterns of polydrug use among ketamine Injectors in New York City. Subst Use Misuse (2005) 40:1381–97 10.1081/JA-200066936 16048823 PMC1899171

[13] Monga N Rehm J Fischer B Brissette S Bruneau J El-Guebaly N Using latent class analysis (LCA) to analyze patterns of drug use in a population of illegal opioid users. Drug Alcohol Depend (2007) 88(1):1–8 10.1016/j.drugalcdep.2006.08.029 17049753

[14] Ramo DE Grov C Delucchi KL Kelly BC Parsons JT Typology of club drug use among young adults recruited using time-space sampling. Drug Alcohol Depend (2010) 107:119–27 10.1016/j.drugalcdep.2009.09.014 19939585 PMC2821995

[15] Sanders B Lankenau SE Jackson-Bloom J Hathazi D Multiple drug use and polydrug use amongst homeless traveling youth. J Ethn Subst Abuse (2008) 7:23–40 10.1080/15332640802081893 19842299

[16] Agrawal A Lynskey MT Madden PA Bucholz KK Heath AC A latent class analysis of illicit drug abuse/dependence: results from the National Epidemiological Survey on Alcohol and Related Conditions. Addiction (2007) 102(1):94–104 10.1111/j.1360-0443.2006.01630.x 17207127

[17] Lynskey MT Agrawal A Bucholz KK Nelson EC Madden PA Todorov AA Subtypes of illicit drug users: a latent class analysis of data from an Australian twin sample. Twin Res Hum Genet (2006) 9(4):523–30 10.1375/twin.9.4.523 16899159

[18] White AM Hingson RW Pan IJ Yi HY Hospitalizations for alcohol and drug overdoses in young adults ages 18–24 in the United States, 1999–2008: results from the Nationwide Inpatient Sample. J Stud Alcohol Drugs (2011) 72(5):774–86 21906505 10.15288/jsad.2011.72.774 PMC3357438

[19] White A Chan GCK Quek LH Connor JP Baker P Saunders JB The topography of multiple drug use among adolescent Australians: findings from the National Drug Strategy Household Survey. Addict Behav (2013) 38(4):2038–73 10.1016/j.addbeh.2013.01.001 23403274

[20] Reyes CR Perez CM Colon HM Dowell MH Cumsille F Prevalence and patterns of polydrug use in Latin America: analysis of population-based surveys in six countries. Rev Eur Stud (2013) 5:10–8 10.5539/res.v5n1p10

[21] Smith GW Farrell M Bunting BP Houston JE Shevlin M Patterns of polydrug use in Great Britain: findings from a national household population survey. Drug Alcohol Depend (2011) 113(2–3):222–8 10.1016/j.drugalcdep.2010.08.010 20863629

[22] McCabe SE Cranford JA Morales M Young A Simultaneous and current polydrug use of alcohol and prescription drugs: prevalence, correlates, and consequences. J Stud Alcohol (2006) 67:529–37 16736072 10.15288/jsa.2006.67.529 PMC1761923

[23] Marshall JE Multiple substance use. Psychiatry (2006) 5(12):461–3 10.1053/j.mppsy.2006.09.005

[24] Schensul JJ Convey M Burkholder G Challenges in measuring concurrency, agency and intentionality in polydrug research. Addict Behav (2005) 30(3):571–4 10.1016/j.addbeh.2004.05.022 15718073

[25] Devlin RJ Henry JA Clinical review: major consequences of illicit drug consumption. Crit Care (2008) 12(1):202 10.1186/cc6166 18279535 PMC2374627

[26] McCabe SE Cranford JA Boyd CJ The relationship between past-year drinking behaviors and nonmedical use of prescription drugs: prevalence of co-occurrence in a national sample. Drug Alcohol Depend (2006) 84(3):281–8 10.1016/j.drugalcdep.2006.03.006 16621337 PMC1706074

[27] Arnett JJ Emerging adulthood – a theory of development from the late teens through the twenties. Am Psychol (2000) 55(5):469–80 10.1037/0003-066X.55.5.469 10842426

[28] Arnett JJ The developmental context of substance use in emerging adulthood. J Drug Issues (2005) 35(2):235–53 10.1016/j.jaac.2010.08.017 21093769 PMC3099425

[29] Humensky JL Are adolescents with high socioeconomic status more likely to engage in alcohol and illicit drug use in early adulthood? Subst Abuse Treat Prev Policy (2010) 5:19 10.1186/1747-597X-5-19 20687935 PMC2924306

[30] Melotti R Heron J Hickman M Macleod J Araya R Lewis G Adolescent alcohol and tobacco use and early socioeconomic position: the ALSPAC birth cohort. Pediatrics (2011) 127(4):e948–55 10.1542/peds.2009-3450 21402626

[31] Briere FN Fallu JS Descheneaux A Janosz M Predictors and consequences of simultaneous alcohol and cannabis use in adolescents. Addict Behav (2011) 36(7):785–8 10.1016/j.addbeh.2011.02.012 21429672

[32] AIHW 2007 National Drug Strategy Household Survey: Detailed Findings. Drug Statistics Series No. 22. Canberra: Australian Institute of Health and Welfare (2008).

[33] Barrett SP Darredeau C Pihl RO Patterns of simultaneous polysubstance use in drug using university students. Hum Psychopharmacol (2006) 21(4):255–63 10.1002/hup.766 16783813

[34] Midanik LT Tam TW Weisner C Concurrent and simultaneous drug and alcohol use: results of the 2000 national alcohol survey. Drug Alcohol Depend (2007) 90(1):72–80 10.1016/j.drugalcdep.2007.02.024 17446013 PMC2043125

[35] Andrews G Slade T Interpreting scores on the Kessler psychological distress scale (K10). Aust N Z J Public Health (2001) 25(6):494–7 10.1111/j.1467-842X.2001.tb00310.x 11824981

[36] ABS Socio-Economic Indices for Areas. Canberra: Australian Bureau of Statistics (2009).

[37] AIHW 2007 National Drug Strategy Household Survey: Detailed Findings. Canberra: Australian Institute of Health and Welfare (2008).

[38] Muthén B Muthén LK Integrating person-centered and variable-centered analyses: growth mixture modeling with latent trajectory classes. Alcohol Clin Exp Res (2000) 24(6):882–91 10.1111/j.1530-0277.2000.tb02070.x 10888079

[39] Schwarz G Estimating the dimension of a model. Ann Stat (1978) 6:461–4 10.1214/aos/1176344136

[40] Sclove SL Application of model-selection criteria to some problems in multivariate analysis. Psychometrika (1987) 52(3):333–43 10.1007/BF02294360

[41] Akaike H A new look at the statistical model identification. IEEE Trans Automat Contr (1974) 19(6):716–23 10.1109/TAC.1974.1100705

[42] Lo Y Mendell NR Rubin DB Testing the number of components in a normal mixture. Biometrika (2001) 88(3):767 10.1093/biomet/88.3.767

[43] StataCorp Stata Statistical Software: Release 11. College Station, TX: StataCorp LP (2009).

[44] Muthen BO Muthen LC Mplus Version 6.01. Los Angeles: Muthen and Muthen (2011).

[45] Nylund KL Asparouhov T Muthén BO Deciding on the number of classes in latent class analysis and growth mixture modeling: a Monte Carlo simulation study. Struct Equ Modeling (2007) 14(4):535–69 10.1080/10705510701575396

[46] Yang C-C Evaluating latent class analysis models in qualitative phenotype identification. Comput Stat Data Anal (2006) 50(4):1090–104 10.1016/j.csda.2004.11.004

[47] Agrawal A Lynskey M Tobacco and cannabis co-occurrence: does route of administration matter? Drug Alcohol Depend (2009) 99(1–3):240–7 10.1016/j.drugalcdep.2008.08.007 18926646 PMC2680145

[48] Higgins ST Rush CR Huges JR Bickel WK Lynn M Capeless MA Effects of cocaine and alcohol, alone and in combination, on human learning and performance. J Exp Anal Behav (1992) 58(1):87–105 10.1901/jeab.1992.58-87 1645103 PMC1322115

[49] Mohamed WMY Hamida SB Cassel J-C de Vasconcelos AP Jones BC MDMA: interactions with other psychoactive drugs. Pharmacol Biochem Behav (2011) 99(4):759–74 10.1016/j.pbb.2011.06.032 21756931

[50] Epstein DH Marrone GF Heishman SJ Schmittner J Preston KL Tobacco, cocaine, and heroin: craving and use during daily life. Addict Behav (2010) 35(4):318–24 10.1016/j.addbeh.2009.11.003 19939575 PMC2849634

[51] Kelly AB Jackson-Carroll CJ Interactivity and equifinality of risks for adolescent smoking. J Child Adolesc Subst Abuse (2007) 17(1):51–64 10.1300/J029v17n01_03

[52] Kelly AB Lapworth K The HYP program – targeted motivational interviewing for adolescent violations of school tobacco policy. Prev Med (2006) 43(6):466–71 10.1016/j.ypmed.2006.06.018 16920183

[53] Grinman MN Chiu S Redelmeier DA Levinson W Kiss A Tolomiczenko G Drug problems among homeless individuals in Toronto, Canada: prevalence, drugs of choice, and relation to health status. BMC Public Health (2010) 10:94 10.1186/1471-2458-10-94 20181248 PMC2841106

[54] Thompson RG Hasin DS Cigarette, marijuana, and alcohol use and prior drug treatment among newly homeless young adults in New York City: relationship to a history of foster care. Drug Alcohol Depend (2011) 117(1):66–9 10.1016/j.drugalcdep.2010.12.020 21288659 PMC3100368

